# Supplementary material for: Reproductive and Obstetric Outcomes after UAE, HIFU, and TFA of Uterine Fibroids: Systematic Review and Meta-Analysis
Source: Int J Environ Res Public Health. 2023 Mar 2;20(5):4480. doi: 10.3390/ijerph20054480 (PMC10001943; doi:10.3390/ijerph20054480)
Supplement: Supplementary file 1 [file ijerph-20-04480-s001.zip › ijerph-2119845-supplementary.pdf]

Table S1. Risk of bias in included non-randomized studies.

Study, Year	Selection of Participants	Classification of Intervention	Measurement of Outcome	Selection of the Reported Results	Missing Data	Overall
**Torre et al.,[37] 2013**	Mild	Mild	Mild	Mild	Mild	Mild
**Torre et al.,[26] 2016**	Mild	Mild	Mild	Mild	Mild	Mild
**Serres-Cousine et al.,[38] 2021**	Moderate	Mild	Moderate	Moderate	Moderate	Moderate
**McLucas,[39] 2013**	Mild	Mild	Mild	Mild	Mild	Mild
**Mara et al.,[40] 2012**	Mild	Mild	Mild	Mild	Mild	Mild
**Redecha et al.,[42] 2012**	Mild	Mild	Mild	Mild	Mild	Mild
**Zou et al.,[43] 2017**	Mild	Mild	Mild	Mild	Mild	Mild
**Wu et al.,[28] 2020**	Moderate	Mild	Mild	Mild	Mild	Mild
**Rodríguez et al.,[44] 2021**	Mild	Mild	Mild	Mild	Mild	Mild
**Qin et al.,[45] 2012**	Mild	Mild	Mild	Mild	Mild	Mild
**Liu et al.,[46] 2018**	Mild	Mild	Mild	Mild	Mild	Mild
**Li et al.,[47] 2017**	Mild	Mild	Mild	Mild	Mild	Mild
**Thiburce et al.,[48] 2015**	Mild	Mild	Mild	Mild	Mild	Mild
**Łoziński et al.,[27] 2019**	Moderate	Mild	Mild	Mild	Mild	Mild
**Verpalen et al.,[49] 2019**	Mild	Mild	Moderate	Moderate	Moderate	Moderate
**Mindjuk et al.,[50] 2014**	Moderate	Mild	Moderate	Moderate	Moderate	Moderate
**Yoon et al.,[51] 2013**	Mild	Mild	Moderate	Moderate	Mild	Moderate
**Froeling et al.,[52] 2013**	Mild	Mild	Mild	Mild	Moderate	Mild
**Toub,[53] 2017**	Mild	Mild	Moderate	Moderate	Mild	Mild
**Lukes and Green,[54] 2020**	Mild	Mild	Mild	Mild	Mild	Mild
**Garza-Leal,[55] 2019**	Mild	Mild	Moderate	Moderate	Mild	Mild
**Christoffel et al.,[56] 2021**	Severe	Moderate	Moderate	Severe	Mild	Severe
**Jiang et al.,[57] 2014**	Mild	Mild	Moderate	Moderate	Mild	Mild
**Brölmann et al.,[58] 2015**	Mild	Mild	Moderate	Moderate	Mild	Mild

Table S2. Risk of bias in included randomized study.

Study, Year	Randomization	Blinding of Participants	Blinding of Outcome Measurement	Missing Data	Overall
**Daniels et al.,[41] 2021**	Mild	Mild	Mild	Moderate	Mild
